# Estrogen receptor mutations and their role in breast cancer progression

**DOI:** 10.1186/s13058-014-0494-7

**Published:** 2014-12-12

**Authors:** Prasanna G Alluri, Corey Speers, Arul M Chinnaiyan

**Affiliations:** 1 Department of Radiation Oncology, University of Michigan Medical School, 1500 E. Medical Center Drive, B2-C445 UH, Ann Arbor, 48109-5010 MI USA; 2 Michigan Center for Translational Pathology, University of Michigan Medical School, 1400 E. Medical Center Drive, 5316 CCC, Ann Arbor, 48109-5940 MI USA; 3 Comprehensive Cancer Center, University of Michigan Medical School, 1400 E. Medical Center Drive, 5316 CCGC 5940, Ann Arbor, 48109-5940 MI USA; 4 Department of Pathology, University of Michigan Medical School, 1301 Catherine, M5240 Med Sci I, Ann Arbor, 48109-5602 MI USA; 5 Department of Urology, University of Michigan Medical School, 1500 E. Medical Center Drive, 3875 TC, Ann Arbor, 48109-5330 MI USA; 6 Howard Hughes Medical Institute, University of Michigan Medical School, 1500 E. Medical Center Drive, 5316 CCC, Ann Arbor, 48109-5940 MI USA

## Abstract

Endocrine therapy is the mainstay of treatment in estrogen receptor-positive breast cancers and significantly reduces disease recurrence and breast cancer-related mortality. However, acquired resistance to therapy has been noted in nearly one-third of women treated with tamoxifen and other endocrine therapies. Mutations in the estrogen receptor have long been speculated to play a role in endocrine therapy resistance but have been rarely detected. However, recent studies utilizing next-generation sequencing on estrogen receptor-positive, metastatic clinical samples have revealed that recurrent *ESR1* mutations are far more frequent than previously thought and may play an important role in acquired endocrine therapy resistance. Here we review recent advances in detection and characterization of *ESR1* mutations in advanced, endocrine therapy-resistant breast cancers.

**Electronic supplementary material:**

The online version of this article (doi:10.1186/s13058-014-0494-7) contains supplementary material, which is available to authorized users.

## Introduction

Acquired resistance to drug treatment is a major problem in cancer therapy. While many mechanisms of drug resistance to chemotherapeutic agents such as efflux, metabolism and inactivation have been described previously, alterations within the drug target have emerged as a dominant resistance mechanism to targeted therapies [1]. A classic example of such acquired resistance is genomic amplification of the androgen receptor (AR) in prostate cancer following treatment with AR antagonists such as bicalutamide [2]. Other examples include acquired mutations in the kinase domain of BCR-ABL1 in chronic myelogenous leukemia patients treated with imatinib [3] and secondary mutations in epidermal growth factor receptor in nonsmall-cell lung cancer patients treated with selective epidermal growth factor receptor inhibitors such as gefitinib [4]. Target-related alterations that induce treatment resistance have also been described in breast cancer. A mutated isoform of Her2 with truncation of the extracellular domain and constitutive kinase activity has been shown to impair trastuzumab binding and promote treatment resistance [5].

The estrogen receptor (ER) belongs to a family of nuclear hormone receptors that act as ligand-activated transcription factors [6]. The binding of ligand induces a conformational change in the receptor, which translocates to the nucleus, binds as a homodimer to specific DNA sequences termed estrogen response elements (ERE) and regulates the transcription of multiple target genes. The domain architecture of the ER includes an N-terminal hormone-independent transactivation domain (AF1), a highly conserved DNA-binding domain that mediates specific recognition of ERE, a hinge domain that separates the DNA-binding domain from the ligand-binding domain (LBD), a LBD that contains the hormone binding pocket, and a second transactivation domain (AF2) in the C-terminus that is activated in response to ligand binding [7]. The etiological role of estrogens in breast cancer is well established and modulation of estrogen signaling remains the mainstay of breast cancer treatment for the majority of breast cancers classified as ER-positive [8]. Several strategies for inhibiting the estrogen axis in breast cancer exist, including: selective ER modulators such as tamoxifen and raloxifene, which act as selective tissue-specific antagonists of ER in the breast [9]; selective ER degraders such as fulvestrant, which promote ER turnover [10]; and aromatase inhibitors such as exemestane (steroidal aromatase inhibitors), anastazole and letrazole (nonsteroidal aromatase inhibitors) – agents primarily used in postmenopausal women with ER-positive breast cancer – which inhibit estrogen biosynthesis [11].

While endocrine therapy has contributed significantly to reduction in disease development, recurrence and breast cancer-related deaths, one-third of women treated with tamoxifen for 5 years have been reported to have recurrent disease within 15 years [12]. Acquired endocrine therapy-resistant disease has thus been estimated to develop in up to one-quarter of all breast cancers [13]. Intense efforts are therefore focused on studying the underlying molecular mechanisms that contribute to endocrine therapy resistance. Multiple growth factor receptor signaling pathways have been implicated in the development of endocrine therapy resistance, including human epidermal growth factor 2, mitogen-activated protein kinase, phosphoinositide 3-kinase/mammalian target of rapamycin, insulin-like growth factor 1 receptor and fibroblast growth factor receptor signaling pathways [14]. It has been speculated for many years that acquired mutations in ER which occur after initiation of hormone therapy may play a role in treatment failure and disease progression. Sluyser and Mester proposed that certain mutations in steroid receptors may result in their ability to bind to DNA in the absence of ligand and may confer hormone independence in cells harboring such mutant receptors [15]. However, reports of acquired mutations in the ER itself have been sparse despite persistent efforts to identify such mutations [16]-[19].

In one of the first reports of acquired *ESR1* mutations in human breast cancers, Fuqua and coworkers in 1997 described a nonsynonymous mutation in Tyr537 (Y537N) in a study of 30 metastatic breast cancer tumors and implicated this mutation in hormone-independent constitutive activation of the ER [18]. Subsequent studies failed to validate this finding, however, probably because most of these studies focused on primary breast tumors instead of metastatic lesions and employed techniques that had low sensitivity for detecting rare mutations in the background of the wild-type alleles. The advent of more sophisticated and sensitive technologies such as next-generation sequencing has aided the search for genomic alterations in ER in response to endocrine therapy, and recent discoveries have renewed interest in *ESR1* mutations as a potential mechanism of endocrine therapy resistance [20].

## Estrogen receptor mutations in breast cancer

Recently, we initiated the clinical sequencing program MI-ONCOSEQ (The Michigan Oncology Sequencing Program) to identify potential actionable genomic alterations in various cancers [21]. As a part of this program, we performed integrative sequencing on 11 metastatic ER-positive breast cancer patients including whole-exome sequencing of the tumor and matched normal tissue, transcriptome sequencing and low-pass whole-genome sequencing, as needed. In addition to several potentially actionable aberrations, we noted nonsynonymous mutations in *ESR1* in six of the 11 patients (ER L536Q in one patient, D538G in two patients and Y537S in three patients) [22]. Interestingly, all mutations localized to the LBD of the ER. Furthermore, all of the index patients had a history of treatment with anti-estrogens (tamoxifen and/or fulvestrant) and aromatase inhibitors. More significantly, when clinical sequencing was performed on pretreatment primary diagnostic tissue from three of the patients for whom the material was available, these mutations were not detected – suggesting that the mutations were acquired following initiation of endocrine therapy. Consistent with this observation, no *ESR1* mutation was found in the pretreatment, primary resection tissue of 390 ER-positive breast carcinoma patients from The Cancer Genome Atlas study [23] or in our cohort of 80 triple-negative breast carcinoma transcriptomes. Similarly, a whole-genome sequencing study of 46 ER-positive, pretreatment breast cancer samples from two neoadjuvant aromatase inhibitor therapy trials [ClinicalTrials.gov:NCT00084396, ClinicalTrials.gov:NCT00265759] did not reveal any *ESR1* mutations [24]. Interestingly, somatic mutations in the LBD of the ER (Y537C and Y537N) were also detected in four of 373 cases of endometrial cancer in The Cancer Genome Atlas database [25]. It is not known whether these patients have a history of endocrine therapy, although it is tempting to speculate that these mutations arose in patients who had concurrent breast cancer, as these patients often are treated with tamoxifen (which is a known risk factor for endometrial cancer) and estrogen deprivation therapy [26].

Our findings of acquired *ESR1* mutations in metastatic, endocrine therapy-resistant breast cancers have been independently corroborated by other groups and have been described previously by Li and colleagues in ER-positive xenografts derived from poor-prognosis, treatment-resistant tumors [27]. In a targeted approach aimed at understanding the genetic basis for acquired hormonal therapy resistance in ER-positive breast cancers, Toy and coworkers surveyed mutations and copy number alterations in 230 commonly mutated genes of tumors from metastatic ER-positive breast cancer patients [28]. The authors found *ESR1* mutations in seven out of 22 tumors that had matched normal DNA and in an additional two out of 14 tumors that did not have matched normal DNA (for a total of 9/36 cases sequenced). All of the patients had been on hormonal therapy for at least 3 months (and an average of 4.9 years) but experienced disease progression while on therapy. Treatment-naïve primary tumor samples were available from two of the nine patients with *ESR1* mutations and no *ESR1* mutations were found in the primary tumor.

To further validate these findings, the authors analyzed primary tumors as well as a subset of metastatic tumors collected after hormonal therapy from patients enrolled in the BOLERO-2 clinical trial [29]. They noted that *ESR1* mutations were present in only 6/183 (3%) primary tumors but in 5/44 (11%) metastatic tumors. The higher rate of *ESR1* mutations in primary tumors (3%) in this study is probably related to the fact that patients enrolled in the BOLERO-2 clinical trial had advanced breast cancer and had disease progression or recurrence after treatment with an aromatase inhibitor (letrozole or anastrozole). The *ESR1* mutations in these primary tumors thus probably arose in response to estrogen deprivation. In contrast, nearly all studies on treatment-naïve, primary breast tumors showed no evidence of *ESR1* mutations. Remarkably, all mutations in *ESR1* in this study also clustered in the same region in the LBD and showed a high degree of overlap to the mutations described in our study, and included V534E, P535H, L536R, Y537C, Y537N, Y537S, D538G, S463P/Y537N, S463P/D538G and Y537S/D538G, suggesting that the selective pressure of estrogen deprivation enriches for activating mutations in the LBD of *ESR1.*

In a study of genomic profiling of 249 (134 ER-positive and 115 ER-negative) tumor specimens from 208 patients, Jeselsohn and colleagues carried out DNA sequencing of 3,230 exons of 182 cancer-related genes and 37 introns of 14 commonly fused genes, including in 37 pairs of matched primary and metastatic breast tumors [30]. Consistent with previous studies, two ER mutations (Y537C and D538G) were reported in this study in metastatic tumors but not in the matched primary tumors. In another metastatic lesion harboring the ER Y537C mutation, the authors were able to sequence tissue from the same metastatic site at two different time points. The initial biopsy obtained before initiation of tamoxifen did not show the mutation while a subsequent biopsy from the same site after 8 years of treatment did. Overall, 16 *ESR1* point mutations were found across 249 specimens and 12 of those were assessed to be somatic in nature. However, the lack of matched normal specimens in this study made it difficult to make definitive determination of the somatic status of the observed mutations. When the authors focused on previously described codon 537/538 mutations in *ESR1*, they found them in nine of 76 (12%) metastatic, ER-positive tumors and in zero of 58 primary ER-positive and zero of 115 ER-negative tumors. Furthermore, the mutation rate was even higher (20%) in heavily pretreated metastatic patients, who on average had received seven lines of previous treatment, including at least two endocrine treatments.

In another study that included 13 tumor samples from Israeli patients with metastatic, ER-positive breast cancer who failed multiple treatments, Merenbakh-Lamin and coworkers performed commercially available genetic analysis by next-generation sequencing of 182 cancer-related genes on DNA extracted from formalin-fixed paraffin-embedded tissue samples and reported ER D538G mutation in five patients (38%) [31]. Interestingly, the biopsies harboring this mutation originated from liver metastases in all of the patients and all patients who tested positive for the mutation had received at least two lines of endocrine therapy for a minimum of 5 years prior to onset of endocrine resistance. Consistent with other studies, the primary tumors did not harbor any mutations in *ESR1*.

In a study aimed at genomic characterization of endocrine therapy-resistant *ESR1* variants in patient-derived xenografts (PDX), Li and colleagues evaluated ER-positive xenografts derived from poor-prognosis, treatment-resistant tumors by monitoring their growth after transplantation into oophorectomized mice [27]. Among six PDX evaluated, only one exhibited estradiol-dependent growth while four demonstrated estradiol-independent growth. The growth of the remaining xenograft was inhibited by estradiol, consistent with paradoxical response of some advanced breast cancers to estradiol. The authors employed RNA-seq analysis of the PDX and identified genomic alterations in four of the xenografts. One alteration included an *ESR1/YAP1* fusion involving the first four exons of the ER (amino acids 1 to 365) and the C terminus of YAP1 (amino acids 230 to 504). Another alteration involved *ESR1* gene amplification with associated high protein levels, with the other two alterations involving mutational events (an ER Y537S mutation and an E380Q mutation).

The discovery of recurrent *ESR1* mutations in endocrine therapy-resistant, metastatic breast cancer spurred interest in functional characterization of these genetic alterations. In our study, we evaluated the response of *ESR1* mutations to estradiol and anti-estrogens in an ERE–luciferase reporter assay system [22]. Interestingly, all *ESR1* mutants showed robust constitutive activation of the ERE reporter, unlike the wild-type *ESR1* that had little activity in the absence of estradiol. The ESR1 mutants did not show significant further increase in ERE reporter activity following stimulation with estradiol. Interestingly, all of the mutants identified in our study showed a dose-dependent inhibitory response to tamoxifen and fulvestrant. These findings argue that the ER mutants arose under selective pressure from estrogen deprivation therapy rather than from anti-estrogen therapy. However, the *ESR1* mutants showed a slightly blunted response to both 4-hydroxytamoxifen and fulvestrant with a twofold to fourfold increase in half-maximal inhibitory concentration values. Similar high constitutive activation of *ESR1* mutants (except S463P) with concomitant increase in the transcript levels of estrogen-responsive genes such as GREB1, MYC, PGR and TFF1 and reduced, but retained, response to anti-estrogens was noted by Toy and coworkers [28]. When gene expression analysis of MCF7 cells in hormone-depleted medium was performed following transfection with the mutant *ESR1* constructs with the highest constitutive activity (Y537S, D538G and S463P/D538G), the mutants demonstrated a unique and shared gene expression pattern that was distinct from the wild-type receptor. These mutants also demonstrated an elevated level of phosphorylation of Ser118 in the AF-1 domain, a post-translational modification previously implicated in increased ligand-independent activity of the ER. The D538G mutant also demonstrated increased interaction with coactivator protein A1B1 when compared with the wild-type protein. These findings were further corroborated by Jeselsohn and colleagues, who also reported constitutive activation of D538G mutant ER in reporter gene assays and increased ligand-independent interaction of *ESR1* D538G mutant with SRC-1, when compared with the wild-type receptor [30]. Furthermore, the D538G mutant demonstrated increased proliferation and migration in MTT and wound healing assays, respectively, compared with the wild-type protein. Additionally, the *ESR1* mutants discovered in the Li and colleagues xenograft study have also been functionally characterized and both *ESR1/YAP1* and ER Y537S mutants showed increased constitutive activation and proliferation in low-estrogen conditions [27]. Finally, when stable MCF7 cell lines with ectopic expression of wild-type, Y537S and D538G mutants were used to establish tumors in nude mice, cells harboring the mutant protein had dramatically increased tumor growth compared with wild-type cells [28]. These findings suggest that mutant ER proteins afforded significant growth advantage to the breast cancer cells.

Interestingly, the structural and functional characterization of Tyr537 mutations preceded their discovery in human cancers. Since Tyr537 resides in close proximity to the region of the ER that is important for ligand-dependent transcriptional function, extensive mutational studies have been carried out to probe its function in estrogen signaling. For instance, Weis and coworkers carried out site-directed mutagenesis of the ER and substituted Y537 with five differing amino acids (alanine, phenylalanine, glutamic acid, lysine and serine), noting that Y537S displayed the highest constitutive activity and was indistinguishable from wild-type ER activity in the presence of estradiol [32]. Furthermore, the extent of the constitutive activity of the mutant receptors strongly correlated with their ability to interact with SRC-1, a known ER coactivator. The Y537S mutant, which showed full constitutive activity in the absence of estradiol, was thus also capable of maximal interaction with SRC-1 in the complete absence of estradiol. Moreover, the interaction with SRC-1 was blocked by anti-estrogens, consistent with findings that these mutant receptors retained sensitivity to tamoxifen and fulvestrant. This study suggested that certain ER mutations may facilitate shift of helix 12 of *ESR1* into a conformation that mimics the ligand-bound active state of the receptor [33]. In fact, structural studies of Y537S mutant by X-ray crystallography have provided evidence for such a conformational change. Nettles and colleagues crystallized the Y537S mutant protein, in complex with an NR box II peptide from the coactivator protein GRIP1, and demonstrated that the mutant protein exists in the canonical agonist conformation with helix 12 folded across helix 3 and helix 11 [34]. Moreover, the mutant Y537S apostructure showed a high degree of similarity to wild-type ER bound to diethylstilbesterol [35], a full ER agonist, confirming that the Y537S mutant mimics the ligand-occupied, active ER conformation.

These studies have shown that Tyr537 hydrogen bonds with Asn348 in the wild-type receptor, resulting in stabilization of the backbone of the helix 11–12 loop and leaving Leu536 in a solvent-exposed position. However, in the Y537S mutant, Ser537 has been shown to establish a hydrogen-bonding interaction with Asp351 resulting in an altered conformation of the helix 11–12 loop and burial of Leu536 in a solvent-inaccessible position. This has been postulated to contribute to constitutive activity of the Y537S mutant protein. Interestingly, the Y537S surface mutation has been shown to have no impact on the structure of the LBD pocket, an observation consistent with functional studies that demonstrated retained sensitivity to anti-estrogens [22],[28]. These structural studies also support the notion that *ESR1* mutations probably arose in response to an estrogen-deprived state rather than anti-estrogen therapy [22].

While an X-ray crystal structure of D538G mutant protein has not been reported in the literature, extensive structural modeling studies have been reported previously [28],[31]. When molecular dynamics simulation studies of the wild-type and D538G mutant proteins were performed in the absence of ligand and bound to a coactivator protein, TIF2, a hydrogen-bonding interaction was noted between the backbone of Gly538 and the side chain of Asp351 that resulted in shifting of G538 towards helix H3 in the mutant receptor that was not present in the wild-type protein [28]. It has been postulated that the Gly538–Asp531 hydrogen bond was enabled by the flexibility of glycine residue owing to its small size, which allows it to adopt backbone conformations that are not accessible for other amino acids. This results in a conformation of the mutant receptor that is similar to the estrogen-bound wild-type receptor and explains its constitutive activity. The structural basis for constitutive activity of other mutant ER proteins is less well understood although biochemical characterization of some of these mutant proteins has been described [36]-[39].

## Conclusion

**Figure 1:**
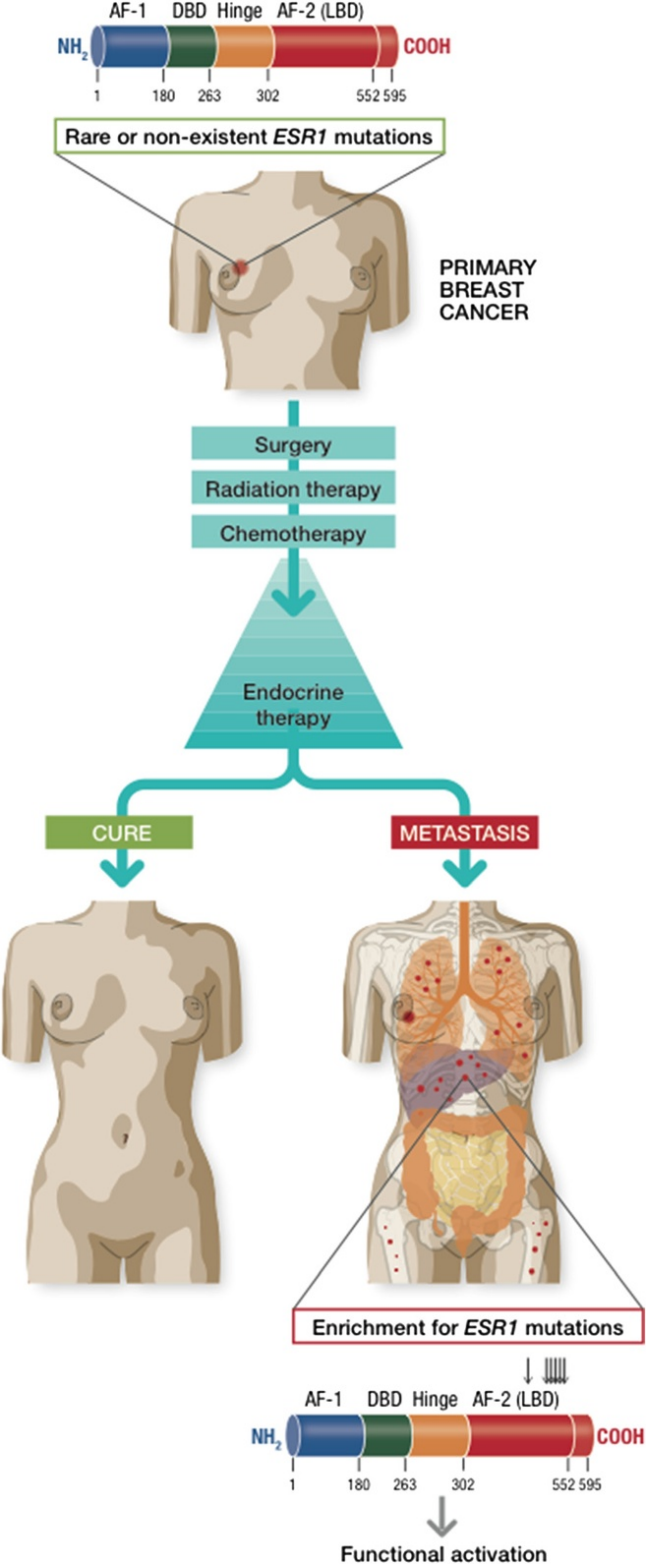
**Evolution of** ***ESR1*** **mutations in estrogen receptor-positive breast cancer with endocrine therapy treatment.** *ESR1* mutations are rare or nonexistent in primary breast tumors and are significantly enriched in metastatic, endocrine therapy-resistant breast cancer. Nearly all *ESR1* mutations localize to the ligand-binding domain (LBD) of the estrogen receptor (ER) and often result in constitutive activation of the ER. DBD, DNA-binding domain.

In summary, *ESR1* mutations are significantly enriched in endocrine therapy-resistant, metastatic breast cancer and are rare or nonexistent in treatment-naïve, primary tumors (Figure 1). Based on published reports, the overall frequency of ER mutations in metastatic, ER-positive breast cancers ranged from 11 to 54.5%, depending on the clinical characteristics of the cohort and the method of identification [22],[28],[30],[31]. Larger prospective studies with standardized detection methods may be needed to establish the true incidence of these mutations. While the evolution of these mutations appears to be strongly correlated with endocrine therapy resistance, a causal relationship between *ESR1* mutations and endocrine therapy resistance remains to be established. We have, however, noted significant upregulation of ER-responsive genes such as GREB1 in tumors harboring *ESR1* mutations, suggesting that ER signaling is active in these tumors and may play a role in conferring endocrine therapy resistance (DR Robinson, AM Chinnaiyan, *et al*., unpublished data).

Future prospective studies involving long-term monitoring of patients undergoing endocrine therapy by less invasive methods such as circulating tumor cells may be necessary to establish the evolutionary timeline of these mutations and their relationship to development of endocrine therapy resistance. Indeed, recent technical advances in single-cell sequencing capability may make future monitoring of *ESR1* mutations in patients receiving endocrine therapy feasible [40]. It remains to be seen whether early detection and intervention, either by a change in endocrine therapy or by addition of other agents, will result in a clinically meaningful improvement in outcomes. In theory, while detection of *ESR1* mutations may prompt a clinician to change the treatment regimen from, say, an aromatase inhibitor to an anti-estrogen, it is already known that patients who develop resistance to aromatase inhibitors often respond to anti-estrogen therapy [41]. Such a change in treatment regimen is thus likely to happen even in the absence of information on *ESR1* mutation status. Whether preemptive changes to the treatment regimen in response to early detection of *ESR1* mutation(s) and prior to onset of clinically detectable disease progression impacts clinical outcomes is an open question and may need to be addressed in a prospective study. Furthermore, the contribution of other genomic and nongenomic alterations that coevolve with *ESR1* mutations also needs to be determined and merits further investigation. Finally, the prognostic significance of *ESR1* mutations in predicting clinical outcomes among patients who develop endocrine therapy resistance remains an area of great clinical interest.

While numerous unknowns remain regarding the role of *ESR1* mutations in advanced, endocrine therapy-resistant breast cancer, their identification certainly opens exciting new avenues of research that will deepen our knowledge and understanding of the molecular basis for acquired endocrine therapy resistance. It is quite remarkable that a very limited number of residues in the LBD of the ER serve as hotspots for evolution of endocrine therapy resistance. It is gratifying that multiple laboratories studying diverse patient populations with advanced, treatment-resistant breast cancer have verified *ESR1* mutations, thus validating those who suggested their existence nearly 30 years ago [15]. These findings have the potential for providing a framework for understanding the structural determinants of ER that contribute to endocrine therapy resistance and coupling them with genomic alterations that evolve in response to therapy.

Furthermore, the retained sensitivity but decreased efficacy of anti-estrogens in preclinical *ESR1* mutant models suggests that the standard dose of anti-estrogens such as tamoxifen often used in the aromatase inhibitor-refractory setting may be inadequate in *ESR1* mutant breast cancers. Prospective clinical trials evaluating the benefits of dose escalation of anti-estrogens such as tamoxifen and fulvestrant may therefore be warranted in *ESR1* mutant breast cancers that have failed standard-dose anti-estrogen therapy. Detailed structural studies on mutant ER proteins may also open the door for development of more potent and mutant-specific ER antagonists and selective ER degraders. Finally, while studies have demonstrated that certain *ESR1* mutations may result in increased interaction with known ER coactivators such as SRC-1 in a ligand-independent fashion, it is unclear whether *ESR1* mutations alter the interactome of the ER. Such studies may uncover novel protein interactions with mutant ERs that may be amenable to therapeutic targeting. In this regard, development of reliable preclinical models of endocrine therapy resistance in the background of *ESR1* mutations would be highly valuable. Encouragingly, the most common *ESR1* mutation (Y537S) in our study as well as in the Chandarlapaty study has independently evolved in PDX grown under low estrogen conditions [27]. It would be interesting to see whether the other *ESR1* alterations detected in the PDX will eventually be found in patient samples. A high concordance between *ESR1* mutations that evolved in PDX and human tumors bolsters our confidence in using PDX as a reliable model for evaluating novel therapeutic strategies for treatment of endocrine therapy resistance that evolved in the background of *ESR1* mutations.

Finally, alterations in the ER that contribute to endocrine therapy resistance provide opportunities for targeting pathways downstream of the ER activation. Recently, we have shown that bromodomain proteins such as BRD4 physically interact with the AR and are necessary for AR-mediated transcription in metastatic castration-resistant prostate cancer models. We have also demonstrated that bromodomain inhibitors such as JQ1 are more efficacious than direct AR antagonism in treatment of castration-resistant prostate cancer in mouse models [42]. These observations provide a further framework for identifying novel effectors of mutant ERs that may serve as attractive targets for therapeutic intervention in advanced, endocrine therapy-resistant breast cancers. The recent discovery of recurrent *ESR1* mutations in metastatic ER-positive breast cancers may merely represent the tip of the iceberg but do provide the basis for further exploration of endocrine therapy resistance mechanisms and the next generation of targeted therapies.

## Authors’ original submitted files for images

Below are the links to the authors’ original submitted files for images. Authors’ original file for figure 1

## Abbreviations

AR: Androgen receptor
ER: Estrogen receptor
ERE: Estrogen response elements
LBD: Ligand-binding domain
PDX: Patient-derived xenografts
