# Supplementary figures and images for: Estrogen receptor mutations and their role in breast cancer progression
Source: Breast Cancer Res. 2014 Dec 12;16:494. doi: 10.1186/s13058-014-0494-7 (PMC4429420; doi:10.1186/s13058-014-0494-7)

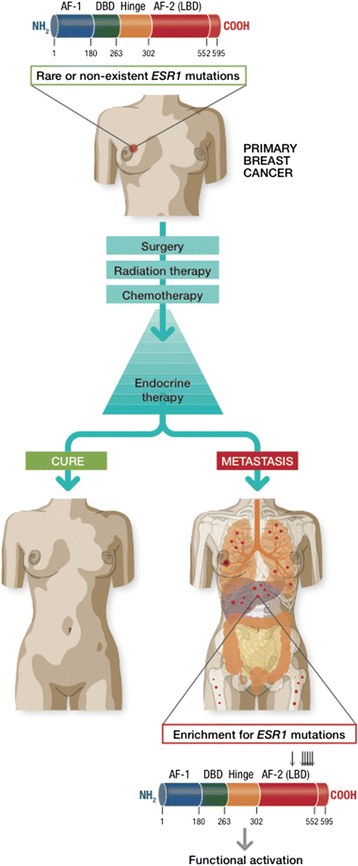

Supplement: Supplementary file 1 — Authors’ original file for figure 1 [file 13058_2014_494_MOESM1_ESM.gif]
